# Post-translational modifications in prion diseases

**DOI:** 10.3389/fnmol.2024.1405415

**Published:** 2024-07-01

**Authors:** Chloé Bizingre, Clara Bianchi, Anne Baudry, Aurélie Alleaume-Butaux, Benoit Schneider, Mathéa Pietri

**Affiliations:** ^1^INSERM UMR-S 1124, Paris, France; ^2^Université Paris Cité, UMR-S 1124, Paris, France; ^3^Ecole polytechnique, Institut Polytechnique de Paris, CNRS UMR7654, Palaiseau, France

**Keywords:** neurodegenerative diseases, signaling, sialylation, phosphorylation, α-Secretases, ROCK, PDK1 (PDPK1), PDK4

## Abstract

More than 650 reversible and irreversible post-translational modifications (PTMs) of proteins have been listed so far. Canonical PTMs of proteins consist of the covalent addition of functional or chemical groups on target backbone amino-acids or the cleavage of the protein itself, giving rise to modified proteins with specific properties in terms of stability, solubility, cell distribution, activity, or interactions with other biomolecules. PTMs of protein contribute to cell homeostatic processes, enabling basal cell functions, allowing the cell to respond and adapt to variations of its environment, and globally maintaining the constancy of the *milieu interieur* (the body’s inner environment) to sustain human health. Abnormal protein PTMs are, however, associated with several disease states, such as cancers, metabolic disorders, or neurodegenerative diseases. Abnormal PTMs alter the functional properties of the protein or even cause a loss of protein function. One example of dramatic PTMs concerns the cellular prion protein (PrP^C^), a GPI-anchored signaling molecule at the plasma membrane, whose irreversible post-translational conformational conversion (PTCC) into pathogenic prions (PrP^Sc^) provokes neurodegeneration. PrP^C^ PTCC into PrP^Sc^ is an additional type of PTM that affects the tridimensional structure and physiological function of PrP^C^ and generates a protein conformer with neurotoxic properties. PrP^C^ PTCC into PrP^Sc^ in neurons is the first step of a deleterious sequence of events at the root of a group of neurodegenerative disorders affecting both humans (Creutzfeldt–Jakob diseases for the most representative diseases) and animals (scrapie in sheep, bovine spongiform encephalopathy in cow, and chronic wasting disease in elk and deer). There are currently no therapies to block PrP^C^ PTCC into PrP^Sc^ and stop neurodegeneration in prion diseases. Here, we review known PrP^C^ PTMs that influence PrP^C^ conversion into PrP^Sc^. We summarized how PrP^C^ PTCC into PrP^Sc^ impacts the PrP^C^ interactome at the plasma membrane and the downstream intracellular controlled protein effectors, whose abnormal activation or trafficking caused by altered PTMs promotes neurodegeneration. We discussed these effectors as candidate drug targets for prion diseases and possibly other neurodegenerative diseases.

## 1 Introduction

The structure, dynamics, and functionalities of proteins depend on or are influenced by post-translational modifications (PTMs), i.e., chemical reactions that occur after the synthesis of proteins. Protein PTMs can be reversible or irreversible and are, most often, driven by enzymes. Reversible PTMs of proteins are associated with the covalent addition of functional or chemical groups, such as the acetyl, phosphate, methyl, glycan, short- and long-chain acyl, or ubiquitin groups, among others, on the side-chain of key amino acids of the targeted protein (Ramazi and Zahiri, 2021). The addition of those chemical functions relies on the activity of specific enzymes (acetylases, kinases, methylases, glycanases, ubiquitinases, etc) that catalyze the transfer of the group from a specific donor to the protein. Those PTMs are reversible as they are removed by hydrolytic enzymes (deacetylases, phosphatases, demethylases, deubiquitinases, etc.) that regenerate a naked protein with functional properties distinct from those of the post-translational modified counterpart. Reversible PTMs contribute to the spatio-temporal regulation of biological processes that notably relate to cell signaling events, genome plasticity, the regulation of gene expression, or energy metabolism (Humphrey et al., 2015; Millán-Zambrano et al., 2022). By contrast, irreversible PTMs of proteins sometimes refer to glycation and deamidation but mostly relate to the proteolytic modifications of proteins. Several proteases achieve the cleavage of the concerned protein, giving rise to protein fragments with distinct biological functions (Walsh et al., 2005). For example, some enzymes (e.g., digestive enzymes) and hormones (e.g., insulin) are synthesized in an immature form, called the pro-form, and turn activated after the proteolytic removal of one or several fragments (Triebel et al., 2022). Those PTMs are irreversible, so the degradation of the modified protein is necessary to regulate the biological process in which the protein is involved. Another type of irreversible, non-classical PTMs relates to the transconformational conversion of proteins, which differs from the subtle protein structural changes associated with protein activity and regulation. The transconformational conversion of proteins consists of deep modifications of the protein folding, i.e., the rearrangement of the protein with changes in the ratio between α-helix and β-sheet elementary folding motifs (Louros et al., 2023). These PTMs that affect the global architecture of the protein cause changes in the physicochemical properties of the protein, such as the surface charges and solubility, thereby leading to protein conformers that often display biological activity distinct from the conformer they derive (Louros et al., 2023). One unfortunate famous example of such post-translational conformational conversion (PTCC) of proteins relates to prions. Prions were highly publicized in the late 20th century with the mad cow disease crisis and the emergence of the variant Creutzfeldt–Jakob disease in humans caused by the transmission of prion pathology from cows to humans through food contaminated with prions (Knight, 2017). Even if the mad cow crisis is behind us, the emergence of chronic wasting disease (CWD) that concerns elk and deer and, for the moment, is confined to North America, Japan, and Scandinavia (Tranulis and Tryland, 2023), combined with the risk of CWD transmission to humans (Hannaoui et al., 2022), necessitates improving our knowledge of the mechanisms underlying prion diseases to rationalize therapeutic strategies to combat these devastating neurodegenerative disorders.

Prion diseases are caused by an infectious and neurotoxic protein, the scrapie protein (PrP^Sc^), which results from the post-translational conformational conversion of the normal cellular prion protein PrP^C^ (Prusiner, 1998). While the 3D structure of PrP^C^ contains three α-helices in the ordered globular domain of the protein, the α-helices are remodeled in favor of the formation of β-sheets in the 3D structure of PrP^Sc^. Such changes in PrP structure confer PrP^Sc^ insoluble properties in detergents and partial resistance to proteolysis. Moreover, PrP^Sc^ β-sheets are responsible for the aggregation of PrP^Sc^ molecules and the formation of fibrillar amyloid assemblies (Cobb et al., 2007; Kraus et al., 2021). As PrP^Sc^ promotes the conversion of PrP^C^ through direct interaction of PrP^Sc^ with PrP^C^, post-translational conformational changes of PrP^C^ into PrP^Sc^ are autocatalytic, which sustains the prion concept formulated in the 80s (Prusiner, 1986). It is established that PrP^C^ conversion into PrP^Sc^ in neurons is at the root of prion diseases (Mallucci et al., 2003). PrP^C^ is a ubiquitous protein that is more expressed in neurons and is present at the cell surface. Acting at the plasma membrane as a neuronal receptor or co-receptor (Mouillet-Richard et al., 2000) or a scaffolding protein that governs the dynamic assembly of signaling modules (Linden et al., 2008), PrP^C^ controls signaling effectors that all contribute to the regulation of neuronal functions (Schneider, 2011). As PrP^C^ is subjected to several PTMs, we here review how those PTMs of PrP^C^ impact the transconformational conversion of PrP^C^ into PrP^Sc^. We also summarize how PrP^C^ PTCC into PrP^Sc^ (i) affects PrP^C^ interactome at the plasma membrane and (ii) impacts PrP^C^ downstream signaling effectors, whose activity or trafficking disturbed by imbalanced PTMs contribute to the progression of prion diseases.

## 2 Post-translational modifications of PrP^C^: friends or foes in prion diseases?

### 2.1 Several PrP^C^ PTMs generate heterogeneity in the PrP^C^ landscape

**Figure 1:**
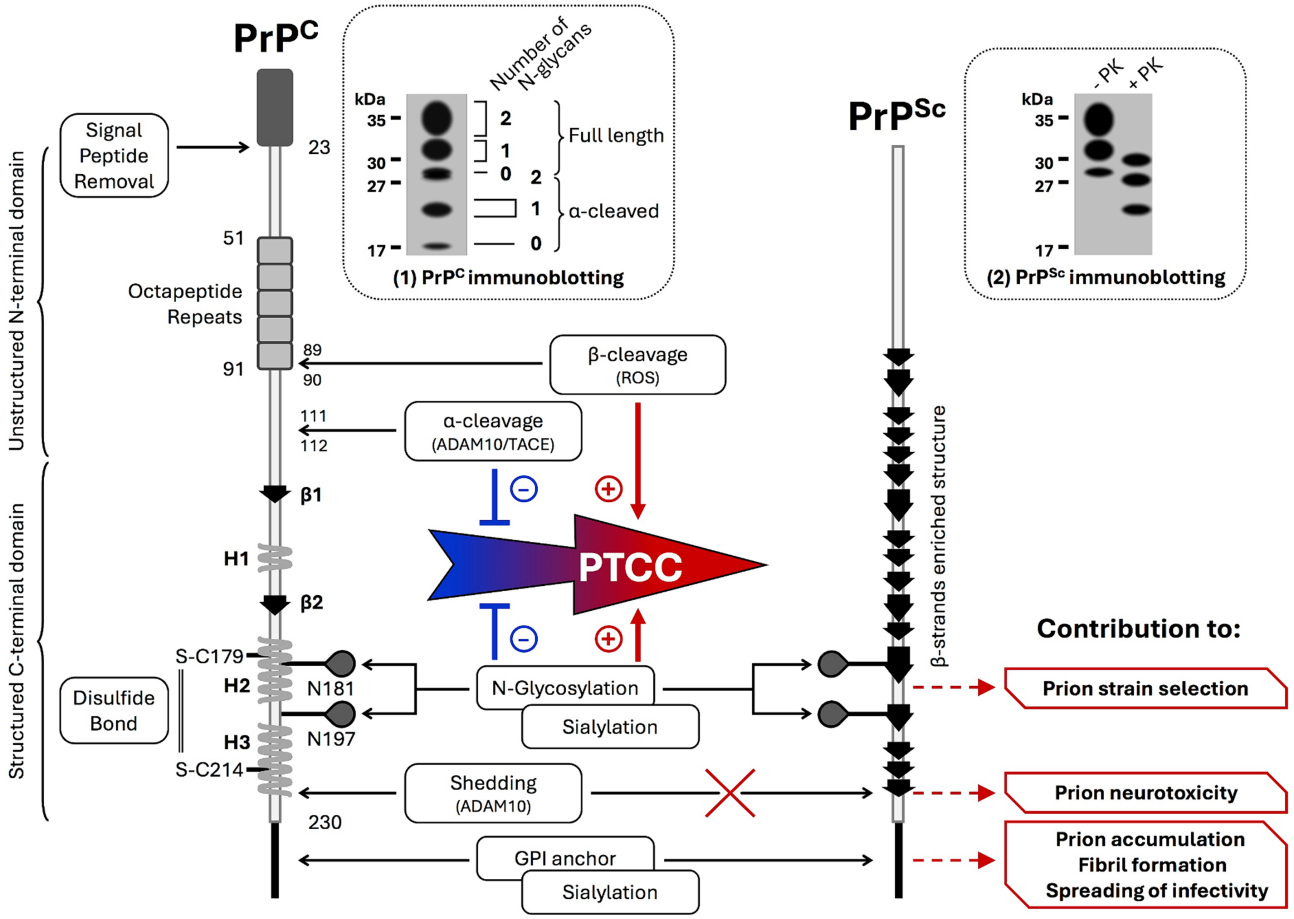
PrP^C^ PTMs influence the post-translational conformational conversion of PrP^C^ into PrP^Sc^ and determine features of prion diseases. Schematic representations of (i) PrP^C^ structure showing PrP^C^ PTMs (glycosylation, sialylation, disulfide bond, GPI anchoring, and α-cleavage) and (ii) PrP^C^ isoforms (non, mono, or biglycosylated full-length and truncated PrP^C^) ranging from 17 to 35 kDa revealed by PrP immunoblotting (insert 1). The post-translational conformational conversion (PTCC) of PrP^C^ into pathogenic prions (PrP^Sc^) changes the 3D structure of PrP^C^ with the suppression of the three α-helices (H1, H2, and H3) and modification of the two β-sheets (β1 and β2) in favor of a global β-sheet enrichment in PrP^Sc^. PrP^Sc^ displays physicochemical properties distinct from PrP^C^, including partial resistance to proteolysis of PrP^Sc^ submitted to proteinase K (PK) digestion (insert 2). Depending on the nature of PrP^C^ PTMs, PrP^C^ PTMs influence, positively or negatively, PrP^C^ PTCC into PrP^Sc^ and take part in prion strain selection, prion accumulation, fibril formation, prion neurotoxicity, and spreading of prion infectivity.

PrP^C^ is coded by the PRNP gene located in chromosome 20 in the human genome (chromosome 2 in mice). The translation of the protein generates a single polypeptide chain of 253 amino-acids that folds as three α-helices and two short anti-parallel β-sheets in the C-terminal PrP^C^ domain, while the N-terminal domain remains flexible (Zahn et al., 2000) and its conformation varies depending on the ligand interacting with this region (Zahn, 2003). Several PTMs occur on PrP^C^ consisting of the removal of the N-terminus signal sequence when the protein enters the endoplasmic reticulum, the attachment of a GlycosylPhosphatidylInositol (GPI) moiety at residue 230 of PrP^C^ C-terminus, two N-glycosylations at Asn181 and Asn197, the formation of a disulfide bridge between Cys179 and Cys214 that ensures the stability of the C-terminal domain of PrP^C^, and the cleavage of the protein between amino-acids 111 and 112 by ADAM10 and TACE (aka ADAM17) α-secretases (Figure 1) (Vincent et al., 2001; Rudd et al., 2002). Such PTMs of PrP^C^ give rise to a heterogeneous population of GPI-anchored PrP^C^ molecules at the plasma membrane, i.e., full-length and truncated PrP^C^ carrying no, one, or two N-glycans (Figure 1). Nevertheless, unglycosylated PrP^C^ represents a minor proportion of PrP^C^ molecules present in the plasma membrane. The nature of the sugars composing the glycans (Ermonval et al., 2009b), which are more or less decorated with sialic acid (Baskakov and Katorcha, 2016), expands the diversity of cell surface PrP^C^ molecules with an apparent molecular mass ranging from 17 to 35 kDa. In cells, sialoglycosylated PrP^C^ resides in lipid rafts of the plasma membrane (Taylor and Hooper, 2006). Sialylation of PrP^C^ GPI anchor acts as a signal that targets the protein to the synapse in neurons (Figure 1) (Bate et al., 2016).

### 2.2 The GPI anchor of PrP^C^ favors the amplification and spread of prion infectivity

As the GPI anchor is found attached to the C-terminus of both PrP^C^ and PrP^Sc^ (Stahl et al., 1990), the GPI anchor has been initially proposed to play an active role in the replication of PrP^Sc^. However, PrP^Sc^ replication occurs when using recombinant PrP^C^ purified from bacteria, i.e., devoid of GPI anchor (Colby et al., 2007). PrP^C^ PTCC into PrP^Sc^ also occurs in mice expressing GPI anchorless-PrP^C^ (Chesebro et al., 2005; Aguilar-Calvo et al., 2017) and in patients with Gerstmann–Sträussler–Scheinker (GSS) Syndrome who express anchorless PrP^C^ due to Q227X stop codon mutation in PRNP gene, i.e., before the site of insertion of the GPI anchor (Shen et al., 2021). Thus, the GPI moiety does not appear to be fundamental to PrP^C^ PTCC into PrP^Sc^. Nevertheless, based on the observation that mice expressing GPI anchorless-PrP^C^ and infected with prions display fibril-containing plaques larger than those in prion-infected wild-type mice, the GPI anchor is suspected to obstruct fibril assembly (Aguilar-Calvo et al., 2017).

Additional experiments combining cell-based assays and *in vivo* approaches also revealed that the GPI anchor is needed for the establishment and maintenance of chronic prion infection within a cell (McNally et al., 2009). Such a role of the GPI anchor in the persistence of prion infection is presumably not attributed to the targeting of PrP^C^ to specific membrane environments compatible with prion formation. GPI-anchored PrP^C^ would rather serve to amplify PrP^Sc^ production, thus enabling the spreading of infectivity between cells (Figure 1) according to different cell modalities (Vilette et al., 2018).

### 2.3 Sialylation of N-glycans of PrP^C^ acts as a filter for prion strain selection

Mutagenesis experiments designed to suppress the two N-linked glycosylation sites in the C-terminal PrP^C^ domain indicated that glycans limit the formation of fibrils and spongiosis in the brains of prion-infected mice (Sevillano et al., 2020). By stabilizing intramolecular interactions with the N-terminal domain of PrP^C^, the N-glycans maintain a PrP^C^ physiological fold that resists the acquisition of a toxic conformation (Schilling et al., 2023). In addition, the sialylation of the N-linked carbohydrates, but not of the GPI anchor, was shown to create a prion replication barrier due to electrostatic repulsion forces between sialic residues that constraint PrP^C^ structure (Katorcha et al., 2015, 2016). Thus, PrP^C^ molecules that are poorly glycosylated with hyposialylated N-linked glycans would be more prone to convert into PrP^Sc^ (Bosques and Imperiali, 2003). Baskakov’s laboratory further showed that the sialoglycan profile of cell surface PrP^C^ dictates the selective recruitment of PrP^C^ molecules by pathogenic prions, leading to the emergence of a prion strain with a unique sialoglycoform signature and prion disease phenotype (Figure 1) (Makarava et al., 2020). Of note, the sialylation state of PrP^Sc^ evolves with PrP^Sc^ invasion of secondary lymphoid organs, such as the spleen, in which PrP^Sc^ is more sialylated than in the brain. The hypersialylation of N-glycans of PrP^Sc^ in secondary lymphoid organs vs. the brain reflects different equipment of sialyltransferases between organs and is proposed as a mechanism that dissimulates PrP^Sc^ from the immune survey (Srivastava et al., 2015). The selection of PrP^C^ molecules with a definite level of sialic acid on N-glycans enters the complex process of prion strain evolution that balances the rapid conversion of PrP^C^ into PrP^Sc^ and PrP^Sc^ protection against the immune system (Makarava and Baskakov, 2023).

### 2.4 The α-cleavage and shedding of PrP^C^ brake PrP^C^ conversion into PrP^Sc^

ADAM10/17-mediated α-cleavage of PrP^C^ between the amino acids 111 and 112 exerts protection against prion infection. Truncated PrP^C^ (also called PrP C1 fragment) resists the post-translational conformational conversion induced by PrP^Sc^ and exerts a dominant negative effect on the conversion of full-length PrP^C^ into PrP^Sc^ (Westergard et al., 2011). However, prion infection cancels PrP^C^ α-cleavage in favor of a redox-induced β-cleavage of PrP^C^ between residues 89/90, generating PrP C2 fragment (Chen et al., 1995). As the C2 fragment converts into PrP^Sc^ and does not inhibit the conversion of full-length PrP^C^ into PrP^Sc^, the PTM switch between the α- and β-cleavage of PrP^C^ contributes to the exponential accumulation of PrP^Sc^. ADAM10 α-secretase also displays the capacity to execute PrP^C^ cleavage upstream of the GPI anchor, which generates a GPI-anchorless PrP called shed PrP (Kovač and Čurin Šerbec, 2018). Shed PrP floats in bodily fluids, binds PrP^Sc^ oligomers, prevents their replication, or acts as nucleation seeds that promote the deposition and neutralization of PrP^Sc^ (Mohammadi et al., 2023). Deficit in ADAM10-mediated shedding of PrP^C^ within a prion infectious context contributes to PrP^Sc^ accumulation and progression of prion diseases (Figure 1) (Chen et al., 2014; Altmeppen et al., 2015).

### 2.5 PTMs of PrP^C^, prion strains, and prion diseases

While PrP^C^ PTCC into PrP^Sc^ is at the root of all prion diseases, these neurodegenerative diseases constitute a heterogeneous group. Considering the same host, prion diseases can exhibit different phenotypes characterized by distinct clinical signs, magnetic resonance imaging (MRI) signals, disease incubation times, brain lesion profiles, and PrP^Sc^ deposit types. These phenotypes are associated with specific PrP^Sc^ biochemical properties in terms of electrophoretic profiles, degree of N-glycosylation, and resistance to proteinase K digestion (Silva et al., 2015). The biochemical and pathological characteristics of PrP^Sc^ are stable and conserved when PrP^Sc^ is successively transmitted to the same host species, leading to the concept of prion strains and their classification into several disease-associated PrP^Sc^ types (Parchi et al., 1999; Collinge and Clarke, 2007; Carta and Aguzzi, 2022). The structure analysis of *ex vivo* pathogenic prions at high resolution by cryogenic electron microscopy (Cryo-Em) revealed that prion strains display analogous β-arch topologies but differ in their conformation details (Kraus et al., 2021; Hoyt et al., 2022; Manka et al., 2022; Cracco et al., 2023). Conformational differences between strains would relate to constraints exerted by PrP^C^ PTMs at the level of N-glycans and the GPI anchor (Vázquez-Fernández et al., 2016).

As mentioned above, the post-translational modifications of PrP^C^ by N-glycosylation, sialylation, and cleavage give rise to a great diversity of PrP^C^ molecules at the cell surface. The PTM profile of PrP^C^ varies according to the cell context due to cell-type specific equipment in sialyltransferases, glycosidases, α-secretases, etc. Specific PTM combinations at the proximal level of PrP^C^ in defined brain areas and peripheral tissues would thus sustain the regio-selective emergence of peculiar prion strains, their accumulation, tropism toward definite neuronal cell types, and the susceptibility of specific neuronal populations to respond to prion strain infection, ultimately leading to neurodegeneration. Supporting the idea of an intricate link between PrP^C^ PTM profiles, regionalized PTCC of PrP^C^ into PrP^Sc^, and prion strain-associated neuropathological lesions, post-mortem detection of spongiform degeneration in sporadic Creutzfeldt–Jakob disease (CJD) brain using diffusion MRI showed that the location of the epicenter and the propagation profile of lesions depend on the prion strain (Pascuzzo et al., 2020). In the near future, single-cell approaches, transcriptomic and proteomic analyses, and the profiling of PrP^C^ PTMs should permit the categorization of brain cell populations that select, replicate, and propagate specific prion strains.

In prion diseases, PrP^C^ PTCC into PrP^Sc^ represents the first critical step in neuropathogenesis, which is, positively or negatively, influenced by a set of limited PTMs that concern the N-glycans, GPI-anchor, and cleavages of PrP^C^ (Figure 1). In the PrP^Sc^-induced neurodegenerative domino game, PrP^C^ PTCC into PrP^Sc^ impacts PrP^C^ signaling partners in the plasma membrane (i.e., PrP^C^ interactome) and downstream PrP^C^-coupled neuronal signaling effectors, whose deregulation causes the death of prion-infected neurons.

## 3 Post-translational modifications of PrP^C^ interactome and prion diseases

### 3.1 PrP^C^ interactome: PrP^C^ orchestrates the organization and activity of membrane signalosomes

Many studies have been conducted to define the interactome of PrP^C^, which currently includes more than 30 protein and non-protein partners. These interactors of PrP^C^ include soluble factors (copper, STI-1, etc.), components of the extracellular matrix (laminin, vitronectin, etc.), or membrane proteins (caveolin 1, NCAM, MARCKS, neuronal receptors, β-integrins, laminin receptor, etc.) (Miranzadeh Mahabadi and Taghibiglou, 2020). The interaction of PrP^C^ with all those partners underlies the complex role of PrP^C^ in membrane signalosomes, i.e., signaling platforms in lipid rafts of the plasma membrane in which PrP^C^ modulates the interactions of cell adhesion molecules with the extracellular matrix or the signaling activity of receptors (Linden et al., 2008; Schneider, 2011). This regulatory role of PrP^C^ in the assembly, dynamics, and activity of membrane signalosomes depends on PrP^C^-induced reversible PTMs of PrP^C^ interactors. Within a prion infectious context, the conversion of PrP^C^ into PrP^Sc^ impacts the PrP^C^ interactome and the PTMs physiologically involved in the regulation of PrP^C^ signalosomes.

### 3.2 PrP^Sc^-induced alteration of PTMs linked to the PrP^C^-caveolin-1 signaling hub disrupts caveolae dynamics and promotes PrP^C^ oversignaling in prion diseases

Our laboratory identified the first PrP^C^-associated signaling platform in neurite extensions of 1C11-derived serotonergic and noradrenergic neuronal cells (Mouillet-Richard et al., 2000). This platform results from the assembly of GPI-anchored PrP^C^ with caveolin-1 (Cav1), a scaffolding protein partially inserted into the inner leaflet of the plasma membrane. Cav1 would interact with the GPI anchor of PrP^C^ via palmitoyl groups present at cysteine residues in the carboxy-terminal domain of Cav1 (Dietzen et al., 1995; Arbuzova et al., 2000). The PrP^C^-Cav1 complex activates the Src tyrosine kinase Fyn on the cytosolic face of the plasma membrane by dephosphorylating the Fyn inhibitory site at Tyr528 (Mouillet-Richard et al., 2000), possibly via the protein tyrosine phosphatase α (PTPα) (Wang et al., 2009). Subsequent phosphorylation of Cav1 at Tyr14 by activated Fyn stabilizes the PrP^C^-Cav1-Fyn platform (Pantera et al., 2009; Gottlieb-Abraham et al., 2013) and initiates downstream intracellular signaling events (Schneider et al., 2003). Depending on the cell type and cell compartment, the PrP^C^-Cav1 complex recruits and activates Fyn but also other tyrosine kinases of the Src family, such as Lyn or Src, in neurons (Lopes et al., 2005; Toni et al., 2006; Caetano et al., 2008), astrocytes (Dias et al., 2016), or immune cells (Stuermer et al., 2004; Krebs et al., 2006).

In addition to its role in the formation and regulation of signalosomes, Cav1 is the major scaffolding protein involved in caveolae formation. Cav1 oligomerization enables the formation of the caveolar coat. The Cav1 phosphorylation or dephosphorylation at Tyr14 destabilizes or stabilizes Cav1 oligomers, respectively, sustaining the bidirectional “kiss-and-run” movement of caveolae between the plasma membrane and the cytosol (Parton et al., 1994; Parton and Simons, 2007; Zimnicka et al., 2016). The Cav1 phosphorylation also drives caveolae anchorage to actin filaments thanks to the interaction between phosphorylated Cav1 and phosphorylated filamin A (Sverdlov et al., 2009). By promoting Cav1 phosphorylation and influencing caveolae movement, PrP^C^ would thus modulate the signaling activity of receptors present in caveolae (Luo et al., 2021).

**Figure 2:**
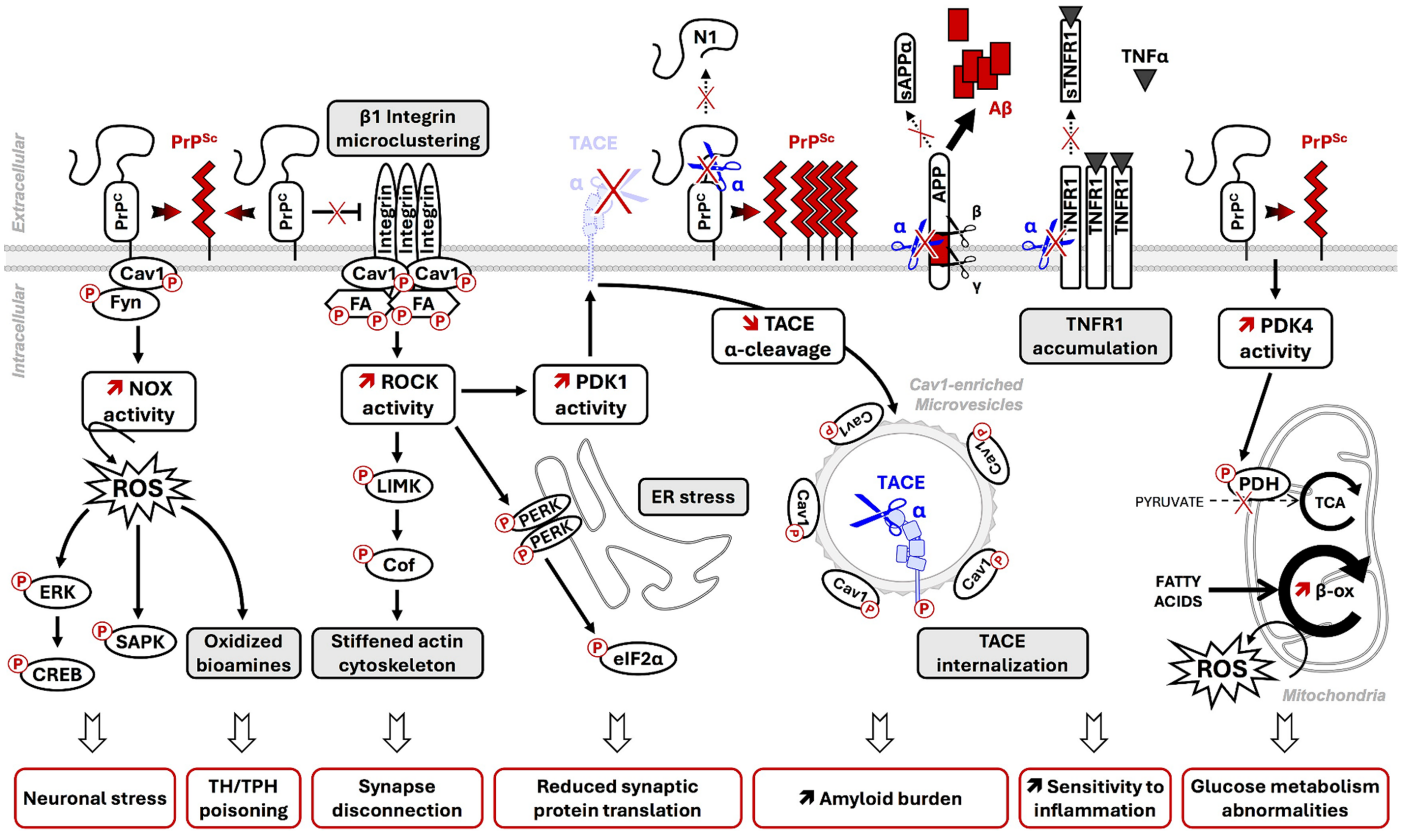
Contribution of PrP^Sc^-induced PTMs dysregulation of PrP^C^ membrane partners and coupled signaling effectors to prion neuropathogenesis. In prion diseases, post-translational conformational conversion (PTCC) of PrP^C^ into PrP^Sc^ alters PTMs of PrP^C^ membrane partners in PrP^C^ signalosomes [Cav1, Fyn, components of focal adhesions (FA)] and deregulates downstream intracellular signaling effectors (NADPH oxidase-NOX, ROCK, PDK1, PDK4). The deregulation of PrP^C^-coupled signaling pathways on PrP^C^ PTCC into PrP^Sc^ contributes to neurodegeneration through multiple deleterious events: onset of oxidative stress conditions, MAPK/SAPK-dependent neuronal stress, alteration of neuronal polarity and functions, amplification of PrP^Sc^ and Aβ production, increased vulnerability to TNFα inflammation by reduced shedding of plasma membrane TNFR into sTNFR, and energy metabolism abnormalities. NOX: NADPH oxidase; ROS: reactive oxygen species; ERK: extracellular-regulated kinases; SAPK: stress-associated protein kinases (p38 and JNK); ROCK: RhoA-associated coiled-coil containing kinases; PERK: PKR-related endoplasmic reticulum kinase; PDK1: 3-phosphoinositide-dependent kinase 1; TACE: TNFα converting enzyme; (s)TNFR: (soluble) TNFα receptor; N1: N-terminal fragment of α-cleaved PrP^C^; α/β/γ: α-, β-, and γ-secretases; sAPPα: neuroprotective α-cleaved APP fragment; Aβ: neurotoxic amyloid β-peptides; PDK4: Pyruvate Dehydrogenase Kinase 4; PDH: Pyruvate Dehydrogenase. TCA: tricyclic acid cycle or Krebs cycle; β-ox: fatty acid β-oxidation.

Within a prion infectious context, co-immunoprecipitation experiments showed that PrP^Sc^ also interacts with Cav1 in brain homogenates of prion-infected hamsters (Shi et al., 2013). Our laboratory provided evidence in 1C11 neuronal cells and mouse brains infected by prions that PrP^Sc^ chronically activates Fyn kinase (Pietri et al., 2006; Pradines et al., 2013) and disrupts the “kiss-and-run” dynamics of caveolae, leading to the accumulation and freezing of Cav1-enriched vesicles underneath the plasma membrane (Figure 2) (Pietri et al., 2013). The freezing of Cav1-enriched vesicles within prion-infected neurons likely reduces the stock of Cav1 available at the plasma membrane and dampens Cav1 regulatory functions in signal transduction, lipid raft-dependent endocytosis, etc. (Cha et al., 2015). We anticipate that, in prion-infected neurons, the sequestration of signalosomes in intracellular freeze caveolae alters the physiological homeostatic activity of signalosomes at the plasma membrane.

Interestingly, the fluidity of the plasma membrane also depends on caveolae fusion with the plasma membrane (Sohn et al., 2018; Yang et al., 2020). Whether, in prion-infected neurons, the freezing of caveolae in the cytosol caused by the excessive phosphorylation of Cav1 at Tyr14 affects the elastic properties of the plasma membrane and thus contributes to neurodegeneration deserves further investigation.

### 3.3 PrP^Sc^-induced alteration of PTMs linked to PrP^C^ interaction with adhesion molecules impacts cell adhesion and neuronal polarity in prion diseases

Although varying with the cell type, the molecular composition of the PrP^C^ interactome always includes several proteins involved in cell adhesion, e.g., extracellular matrix proteins, such as laminin, vitronectin, and fibronectin, and membrane proteins, including integrins, neural cell adhesion molecule (NCAM), and myristoylated alanine-rich C-kinase substrate (MARCKS) proteins (Ghodrati et al., 2018). MARCKS are proteins inserted into the inner leaflet of the plasma membrane via a positively charged domain and a myristoyl group added to Gly2 (Arbuzova et al., 2000). These proteins likely interact with the GPI anchor of PrP^C^.

A global proteomic study comparing PrP^C^ interactomes of four mouse cell lines and the mouse brain revealed a highly conserved functional relationship between PrP^C^, MARCKS, and NCAM1 (Mehrabian et al., 2016). PrP^C^ directly interacts with NCAM1, promoting NCAM1 recruitment to lipid rafts and NCAM1-dependent Fyn kinase activation (Santuccione et al., 2005; Lehembre et al., 2008). Through its coupling to MARCKS, PrP^C^ indirectly controls NCAM1 sialylation by regulating the expression of the polysialyltransferase ST8SIA2 (Boutin et al., 2009; Mehrabian et al., 2015). The PrP^C^-induced increase in NCAM1 polysialylation limits homophilic and heterophilic NCAM1 interactions and thereby modulates cell adhesion, a critical event for the onset and maintenance of neuronal polarity (Mehrabian et al., 2016). Any PrP^Sc^-induced disturbance of the PrP^C^-NCAM1-MARCKS signalosome would affect neuronal polarity in prion diseases.

Beyond NCAM1, PrP^C^ is also functionally involved in the regulation of other adhesion proteins, such as β1 integrins, which orchestrate the assembly and the turnover of focal adhesions (FAs). PrP^C^ prevents β1 integrin microclustering and attenuates β1 integrin signaling in 1C11 and PC12 cells (Loubet et al., 2012). By interacting with β1 integrins, PrP^C^ would block the structural modifications required for β1 integrin activation. Alternatively, PrP^C^ would limit β1 integrin signaling by neutralizing some β1 integrin activators such as CD98 or thrombospondin-1 (Ghodrati et al., 2018). In prion-infected neuronal cells (Alleaume-Butaux et al., 2015), as well as in the brains of mice infected with the Chandler or Rocky Mountain Laboratory (RML) prion strains (Albert-Gasco et al., 2024), PrP^Sc^-induced depletion of plasma membrane PrP^C^ causes a loss of PrP^C^’s regulatory role toward β1 integrins. This depletion leads to β1 integrin microclustering and oversignaling (Alleaume-Butaux et al., 2015). β1 integrin oversignaling induced by PrP^Sc^ likely promotes chronic Src kinase phosphorylation at Tyr418 and activation, the subsequent phosphorylation of focal adhesion kinase (FAK) at Tyr861 by Src kinases, and the phosphorylation of paxillin at Tyr31 and Tyr118 by both Src kinases and FAK, as observed in cells depleted for PrP^C^ (Loubet et al., 2012; Alleaume-Butaux et al., 2015; Ezpeleta et al., 2017). Such an overphosphorylated state of FA components enhances FA stability and reduces FA dynamics that disturb the adhesion properties of prion-infected neurons. This mechanism would contribute, at least in part, to the loss of neuronal polarity on prion infection (Figure 2) (Alleaume-Butaux et al., 2015).

Finally, PrP^C^ modulates the laminin-mediated attachment of neurons to the extracellular matrix. PrP^C^ interacts with laminin and laminin receptors (LRs) and limits laminin binding to its membrane receptor (Baloui et al., 2004). Our laboratory further showed in 1C11 neuronal cells that PrP^C^, through its coupling to Cav1 and Fyn, regulates the activity of tissue non-specific alkaline phosphatase (TNAP), which phosphorylates laminin and reduces laminin-dependent adhesion of cells (Ermonval et al., 2009a). As PrP^Sc^ interacts with LR in neuronal cells (Morel et al., 2005) and overactivates the PrP^C^-Cav1-Fyn platform, the excessive phosphorylation of laminin induced by PrP^Sc^ would also destabilize the neuronal cell-matrix interaction and neuronal polarity in prion-infected neurons.

Thus, by affecting several homeostatic PTMs in the PrP^C^ adhesion interactome, PrP^Sc^ alters cell–cell and cell-extracellular matrix contacts required for the onset and stability of the neuronal polarity and the neuronal plasticity.

### 3.4 PrP^Sc^-induced alteration of PTMs linked to PrP^C^ interaction with synaptic protagonists disturbs neurotransmission in prion diseases

At the presynaptic and post-synaptic membrane of neurons, PrP^C^ interacts with several ionotropic (NMDAR, AMPAR GluA1/2, KAR GluR6/7, and α7nAChR) and metabotropic (mGluR1/5) receptors (Kleene et al., 2007; Beraldo et al., 2010, 2011; Carulla et al., 2011; Watt et al., 2012; Um et al., 2013).

Several studies have highlighted that group I metabotropic glutamate receptors (mGluR1/5) act as co-receptors of PrP^C^. The binding of laminin or STI1 to PrP^C^ activates PrP^C^, which in turn recruits and activates mGluR1/5 (Coitinho et al., 2006; Beraldo et al., 2011). mGluR1/5 are G protein-coupled receptors (GPCR) coupled to heterotrimeric Gαq/11 proteins involved in glutamate-dependent memory consolidation through neuritogenesis and neuronal plasticity events. The molecular mechanisms underlying PrP^C^ modulation of mGluR1/5 activity remain, however, elusive. Nevertheless, the beneficial effect of mGluR1/5 inhibition in scrapie-infected mice suggests that PrP^Sc^ impacts the PrP^C^-mediated regulation of mGluR1/5 activity (Goniotaki et al., 2017). Since activation of mGluR1/5 involves binding of Fyn kinase to the C-terminal domain of mGluR1/5 and Fyn-dependent phosphorylation of mGluR1/5 at Tyr937 (Jin et al., 2017), chronic activation of Fyn by PrP^Sc^ may corrupt mGluR1/5 activity by imbalanced phosphorylation of mGluR1/5.

NMDAR is a calcium-permeable channel composed of two GluN1 and two GluN2 or GluN3 subunits involved in glutamate-mediated neuronal plasticity and excitotoxicity. PrP^C^ interacts with GluN2D and GluN2B subunits (Khosravani et al., 2008; Barnes et al., 2020) and controls NMDA receptor activity through nitrosylation of GluN1 and GluN2A subunits (Gasperini et al., 2015). PrP^C^-bound Cu^2+^ acts as an electron acceptor that induces NO oxidation and subsequent S-nitrosylation of two cysteines on GluN1 and three cysteines on GluN2A, including Cys399, which mediates the predominant inhibitory effect on NMDAR activity. The reduction of NMDAR S-nitrosylation in prion-infected mice before the onset of clinical signs increases NMDAR-dependent excitation (Ratté et al., 2008). PrP^Sc^ also induces phosphorylation of the NMDAR GluN2B subunit at Tyr1472, probably via sustained activation of Fyn, and potentiates NMDAR activity in the hippocampus of a mouse model of CJD (Bertani et al., 2017). By decreasing S-nitrosylation and increasing phosphorylation of NMDAR, PrP^Sc^ enhances NMDAR activity and renders prion-infected neurons hypersensitive to NMDA-induced excitotoxicity (Meneghetti et al., 2019). Disturbance of PrP^C^-governed PTMs of neuronal receptors by PrP^Sc^ thus alters neurotransmission in prion diseases.

Of note, the synapse interactome of PrP^C^ also includes synapse-associated proteins (synaptophysin and PSD-95), vesicle-associated proteins (synapsin), and ion pumps (Kv4.2 DPP6 and VGCC). Through these interactions, PrP^C^ contributes to the assembly of functional complexes involved in neurotransmission at the pre- and post-synaptic membranes. Further investigations are needed to assess whether PrP^Sc^-mediated disruption of those complexes or imbalanced PTMs of the above-mentioned synapse effectors would also contribute to the alteration of neurotransmission in prion diseases (Russelakis-Carneiro et al., 2004).

## 4 PTMs of signaling effectors downstream of PrP^C^ in prion diseases

### 4.1 PrP^C^ contribution to neuronal homeostasis depends on PrP^C^ coupling to several signaling effectors

The use of diverse neuronal cell lines (N2a neuroblastoma cells, 1C11 neuronal stem cells and their serotonergic or noradrenergic neuronal progenies, PC12 pheochromocytoma cells, etc.) and primary cultures of neurons (cerebellar granule neurons, cortical or hippocampal neurons) showed that PrP^C^ is involved in the regulation of a wide range of cellular functions, including cell adhesion (see Section 3.3), neuronal differentiation, synaptic plasticity, cell survival, redox equilibrium, stress protection, or energy metabolism (Schneider, 2011; Castle and Gill, 2017; Wulf et al., 2017; Schneider et al., 2021). This multifaceted role of PrP^C^ involves its capacity to act as a receptor/co-receptor, governing a complex signaling network, and as a scaffolding protein that regulates the lipid rafts of the plasma membrane. This regulation affects the assembly and stoichiometry of interaction between partners such as integrins, laminin receptors, and mGluR, thereby controlling the activity of diverse signaling modules. Regardless of the context, PrP^C^ is coupled to several intracellular signaling effectors, including Src kinase Fyn, NADPH oxidase, ERK1/2 MAP kinases, glycogen synthase kinase 3β (GSK3β), Protein kinase A (PKA), RhoA-associated coiled-coil containing kinases (ROCKs), 3-phosphoinositide-dependent kinase 1 (PDK1), Pyruvate Dehydrogenase Kinase 4 (PDK4), TACE α-secretase (aka ADAM17), and CREB transcription factor. These effectors all play a part in maintaining the homeostasis of neuronal functions (Chiarini et al., 2002; Hernandez-Rapp et al., 2014; Arnould et al., 2021; Schneider et al., 2021). For most of the signaling intermediates downstream of PrP^C^, their regulation depends on reversible and transient PTMs by phosphorylation, which sustains the fine-tuning of neuronal functions. Within a prion infectious context, the corruption of PrP^C^ signaling in response to PrP^C^ PTCC into PrP^Sc^ leads to imbalance PTMs of PrP^C^-coupled signaling effectors, thus generating aberrant and deleterious signals for prion-infected neurons (Figure 2). A global comparative phospho-proteome analysis between PrP^Sc^-infected N2a cells and non-infected cells identified 105 proteins differentially phosphorylated with chronic and excessive phosphorylation of some effectors (e.g., cofilin) or loss of phosphorylation for some others (e.g., stathmin) (Wagner et al., 2010). We here review PTMs of some PrP^C^-coupled signaling effectors affected by prion infection and the consequences thereof for neurons.

### 4.2 PrP^Sc^-induced corruption of PrP^C^/Fyn/NADPH oxidase signaling causes the recruitment of stress-sensitive SAPK and the accumulation of bioamine-derived neurotoxins

One signaling pathway impacted by PrP^Sc^ in prion-infected neurons is the PrP^C^/Fyn/NADPH oxidase cascade. The chronic stimulation of this pathway triggers excessive production of reactive oxygen species (ROS) by NADPH oxidase (NOX) at the root of oxidative stress conditions. Overproduced ROS promote robust phosphorylation of MAPKs ERK1/2 (at Thr185/Tyr187) and additionally recruit stress-associated protein kinases (SAPKs) p38 and JNK1/2, which are activated by phosphorylations at Thr180/Tyr182 and Thr183/Tyr185, respectively. The subsequent sustained activation of MAPKs and SAPKs contributes to the death of prion-infected neurons by apoptosis (Figure 2) (Pietri et al., 2006; Pradines et al., 2013). The local rise of p38 phosphorylation at Thr180 and Tyr182 in dendritic spines of prion-infected hippocampal neurons was also shown to promote synaptic degeneration and decrement in synaptic transmission (Fang et al., 2018). Augmented phosphorylation of ERK1/2, p38, and JNK was confirmed *in vivo* in the brains of hamsters infected with the 263 K prion strain (Lee et al., 2005; Pamplona et al., 2008). Of note, downregulating this pathway through the use of siRNAs against Fyn or the p22phox subunit of NADPH oxidase reverts those PTMs on ERK1/2, p38, and JNK induced by prion infection, supporting the therapeutic potential of targeting Fyn or NADPH oxidase to protect neurons in prion diseases (Pradines et al., 2013).

In addition, ROS overproduced by NADPH oxidase in prion-infected serotonergic or noradrenergic neurons promote the generation of oxidized derivatives of serotonin (5-HT), such as tryptamine 4,5-dione (T-4,5-D) and 5,6-dihydroxytryptamine (5,6-DHT), or catecholamines, such as 6-hydroxydopamine and tetrahydroisoquinolines. These derivatives are considered neurotoxins (Mouillet-Richard et al., 2008). Their neurotoxic action relies on the poisoning of metabolic enzymes involved in the synthesis of 5-HT or noradrenalin (NE), that is, a set of “toxic and accidental PTMs” affecting the tryptophan hydroxylase TPH (the rate-limiting enzyme for 5-HT synthesis) and possibly the tyrosine hydroxylase TH (the rate-limiting enzyme for NE synthesis) through the covalent grafting of 5-HT/catecholamine-derived neurotoxins at catalytic Cys residues of those biosynthetic enzymes (Figure 2).

Another consequence of ERK1/2 activation is the downstream activation of the transcription factor CREB by phosphorylation at Ser133 (Figure 2) (Lee et al., 2005; Pradines et al., 2013). In prion-infected neurons, sustained CREB phosphorylation stimulates the expression of the immediate-early genes Egr-1 implicated in cell survival but lockdowns the transcription of the MMP9 encoding gene, which attenuates the metalloproteinase activity of MMP9. The decrease in MMP9 enzymatic activity leads to a reduction of β-dystroglycan cleavage at the neuronal cell surface that alters the interactions between neurons and the extracellular matrix (Pradines et al., 2013). Such CREB PTMs and subsequent modifications of gene expression in prion-infected neurons would contribute to the alterations of neuronal plasticity associated with prion diseases.

### 4.3 Rock oversignaling upon prion infection alters neuronal polarity and takes part in the unfolded protein response

Our study documented that PrP^Sc^ abrogates the negative regulatory role exerted by PrP^C^ on ROCK signaling due to a loss of PrP^C^ control of β1 integrin microclustering and signaling activity (Alleaume-Butaux et al., 2015). In prion-infected 1C11 neuronal cells, N2a58 neuroblastoma cells, mouse cerebellar granule neurons, hippocampal neurons, and the brains of prion-infected mice, ROCK overactivity leads to excessive phosphorylation of LIMK1/2 at Thr505 and Thr508 and cofilin at Ser3. Stable phosphorylation of cofilin in prion-infected neurons cancels the severing activity of cofilin on the actin cytoskeleton (Figure 2) (Wagner et al., 2010; Alleaume-Butaux et al., 2015; Kim et al., 2020). The resulting PrP^Sc^-induced stiffening of the actin cytoskeleton associated with fewer dynamics of focal adhesions (see section 3.3) disrupts neuronal polarity and provokes synaptic disconnection and dendrite/axon degeneration (Alleaume-Butaux et al., 2015; Kim et al., 2020). Such neuronal damages are counteracted by ROCK inhibition with pharmacological compounds (Alleaume-Butaux et al., 2015). Alterations in ROCK, LIMK1, and cofilin were also evidenced in the post-mortem cortex and cerebellum samples of sporadic Creutzfeldt–Jakob disease (sCJD) patients at clinical and pre-clinical stages (Zafar et al., 2018), paving the road for developing therapeutic strategies targeting ROCK to limit neurodegeneration in prion diseases.

Apart from the action of ROCK on the actin cytoskeleton, our laboratory showed that overactivated ROCK plays a role in the unfolded protein response (UPR). In 1C11-derived serotonergic neurons infected by mouse-adapted human GSS prions (Fukuoka strain), overactivated ROCK enhances the phosphorylation of PERK at Thr980 in the endoplasmic reticulum. Phosphorylated PERK, in turn, promotes the hyperphosphorylation of the translational initiation factor eiF2α at Ser51 (Schneider et al., 2021), which halts the translation of some proteins involved in the maintenance of synaptic connections (Moreno et al., 2012), likely contributing to synapse failure in prion-infected neurons (Figure 2). It remains unknown whether PERK is a direct substrate of ROCK. In any case, ROCK inhibition decreases the phosphorylation of PERK and eiF2α (Schneider et al., 2021), allowing restarting the expression of synaptic proteins and preserving neuronal transmission within a prion-infectious context.

### 4.4 PrP^Sc^-Induced deregulation of the PrP^C^/PDK1/TACE signaling axis renders prion-infected neurons highly vulnerable to inflammation and amplifies the production of PrP^Sc^ and Aβ

In 2013, our laboratory provided prime evidence that the corruption of PrP^C^ coupling to 3-phosphoinositide-dependent kinase 1 (PDK1) and downstream TACE α-secretase plays a critical role in the neuropathogenesis of prion diseases (Pietri et al., 2013). In prion-infected neurons, overactivated PDK1 promotes TACE phosphorylation at Thr735 and TACE displacement from the plasma membrane to caveolin-1-enriched microvesicles, which neutralizes TACE neuroprotective shedding activity (Figure 2). As PDK1 only admits AGC kinases as substrates (Leroux and Biondi, 2023), it is unlikely that TACE phosphorylation results from a direct action of PDK1 on TACE. The phosphorylation of TACE at Thr735 would be part of signals that impact the subcellular localization of TACE and modulate its shedding activity. Internalized, phosphorylated TACE in prion-infected neurons becomes uncoupled from three major substrates: (i) TNFα receptors (TNFR), which accumulate at the cell surface, rendering neurons hypersensitive to TNFα, (ii) PrP^C^, where the loss of PrP^C^ PTM by α-cleavage between amino-acids 111/112 strongly reduces the C1 fragment of PrP^C^ in favor of full-length PrP^C^ (and C2 fragment), which is highly prone to converting into PrP^Sc^, and (iii) the amyloid precursor protein (APP), where the loss of APP PTM by α-cleavage in favor of APP PTM by β- and γ-secretases leads to the accumulation of neurotoxic Aβ peptides (Figure 2) (Pietri et al., 2013; Ezpeleta et al., 2019). Importantly, inhibiting PDK1 suppresses TACE phosphorylation at Thr735. The subsequent redirection of TACE at the plasma membrane allows TACE to reintegrate cell surface signalosomes and recover its protective shedding activity. This activity includes the cleavages of TNFR, PrP^C^, and APP. Such restored irreversible PTMs of TNFR, PrP^C^, and APP protect prion-infected neurons from TNFα toxicity and limit the production of toxic amyloids (Pietri et al., 2013; Ezpeleta et al., 2019).

PDK1 activity is known to be governed by several events: (i) translocation to the plasma membrane, (ii) post-translational modifications by phosphorylation, (iii) conformational changes, and (iv) interaction with different effectors (Sacerdoti et al., 2023). We demonstrated that the overactivation of PDK1 in prion-infected neurons depends on the upstream kinase ROCK (Alleaume-Butaux et al., 2015). ROCK interacts with PDK1 and promotes phosphorylation of PDK1, a PTM that accounts for the increase in PDK1 enzymatic activity within a prion infectious context. Of note, ROCK-dependent phosphorylation of PDK1 occurs only after autophosphorylation of PDK1 at Ser241. The additional phosphorylation of PDK1 by ROCK leads to sustained PDK1 activity in prion-infected neurons. Importantly, as the pharmacological inhibition of PDK1 or ROCK reduces motor impairment, lowers brain PrP^Sc^ and Aβ levels, and prolongs the lifespan of prion-infected mice, PDK1 and ROCK are currently considered as potential therapeutic targets to combat prion diseases (Pietri et al., 2013; Alleaume-Butaux et al., 2015; Ezpeleta et al., 2019). Therefore, any strategies aiming at rescuing the α-cleavage or shedding of PrP^C^ and APP should help to limit the production of PrP^Sc^ and Aβ and thereby mitigate prion diseases (Linsenmeier et al., 2021).

### 4.5 PrP^Sc^ Deviates the energy metabolism by altering the PrP^C^/PDK4 coupling

We evidenced that prion infection also cancels the regulatory role of PrP^C^ on glucose metabolism, leading to a metabolic reprogramming of infected neurons, i.e., a conversion from glucose oxidative degradation to β-oxidation of fatty acids (Arnould et al., 2021). From a mechanistic point of view, PrP^Sc^ abrogates the negative control exerted by PrP^C^ on the expression of Pyruvate Deshydrogenase Kinase 4 (PDK4) encoding gene, causing a high rise in PDK4 enzymatic activity. By phosphorylating the mitochondrial Pyruvate Dehydrogenase (PDH) complex, overactivated PDK4 decreases the activity of PDH that normally ensures the transfer of cytosolic pyruvate in the mitochondria and its conversion into acetylCoA for the production of energy. The consequences of such PTM of PDH and subsequently reduced activity of PDH are a slowdown of the glycolytic flux and limited oxidative degradation of glucose. To compensate for energy restriction, prion-infected neurons divert their metabolism toward fatty acids β-oxidation. Since fatty acids can act as pro-oxidant molecules, the oxidative stress resulting from the degradation of fatty acids by the β-oxidation pathway has been shown to contribute to neurodegeneration in prion diseases (Figure 2). Interestingly, pharmacological inhibition of PDK4 with dichloroacetate (DCA), a medicine approved for treating congenital lactic acidosis, restores, at least partly, PDH activity in the brains of prion-infected mice, which favors the recovery of glucose metabolism over fatty acids β-oxidation and extends the lifespan of DCA-treated prion-infected mice (Arnould et al., 2021).

### 4.6 PrP^Sc^ alters Ca^2+^ signaling downstream of PrP^C^ interacting neuronal receptors

PrP^Sc^-induced dysregulation of ionotropic (NMDAR) and metabotropic receptors (mGluR) increases the intracellular Ca^2+^ level (Hu et al., 2022). Exposure of mouse cerebellar granule neurons to the neurotoxic PrP amyloidogenic polypeptide (PrP90-231) increases NMDAR-dependent uptake of Ca^2+^ (Thellung et al., 2017). In prion-infected SMB-S15 cells, mGluR oversignaling enhances the release of Ca^2+^ from ER in response to PLC-mediated hydrolysis of 4.5-biphosphate phosphatidylinositol and subsequent IP3 increase, combined with an increased level of IP3 receptor in the ER (Hu et al., 2022). Calmodulin (CaM), a transducer of Ca^2+^ signals that activates different kinases, is upregulated in the cortex of sporadic CJD (sCJD) patients and in the brains of prion-infected hamsters (Shi et al., 2015; Zhang et al., 2017). On prion infection, the upregulation of the Ca^2+^/calmodulin complex increases the Ca^2+^/calmodulin-dependent calcineurin (CaN) phosphatase activity. CaN is a type 2 phosphatase highly expressed in neurons, physiologically involved in synaptic plasticity, memory, and neuronal death. In prion-infected neurons, CaN dephosphorylates the pro-apoptotic protein Bad at Ser112, causing Bad translocation in mitochondria and the subsequent release of cytochrome c from mitochondria to the cytoplasm where it activates caspase-dependent apoptosis pathways (Agostinho et al., 2008). PrP^Sc^-induced CaN overactivity would also alter synaptic plasticity and trigger neurodegeneration by dephosphorylating the substrate slingshot 1 (SHH1) in prion diseases. SHH1 activates cofilin and triggers the formation of cofilin-actin rods, which are involved in glutamate-mediated excitotoxicity (Bamburg et al., 2021). Pharmacological inhibition of CaN with the immunosuppressive drug FK506 limits neurodegeneration, reduces motor deficits, and increases the survival of mice infected with RML or Fukuoka-1 strains (Mukherjee et al., 2010; Nakagaki et al., 2013), introducing an additional way of therapeutic intervention for prion diseases.

The Calpain non-lysosomal cysteine proteases are other enzymes dysregulated by Ca^2+^ overload in prion-infected neurons (Baudry and Bi, 2024). In a sCJD mouse model, prion infection downregulates the neuroprotective Calpain-1 and upregulates the neurodegenerative Calpain-2, leading to global Calpain overactivity in the brain. Overactivated pathological Calpain- 2 enhances the cleavage of Calpain substrates such as Neurofilament Light Chain and γ-tubulin, additionally contributing to the loss of neuronal polarity (Llorens et al., 2017).

## 5 Conclusion

In prion diseases, the initial aberrant PTM concerns normal cellular prion protein PrP^C^ with the post-translational conformational conversion (PTCC) of PrP^C^ into pathogenic prions PrP^Sc^. This dramatic change in PrP^C^ folding is influenced by several PrP^C^ PTMs (glycosylation, sialylation, cleavages, etc.) that oppose or favor the production of PrP^Sc^. One consequence of the conversion of PrP^C^ PTCC into PrP^Sc^ is the deregulation of PTMs at the proximal level of PrP^C^ partners in PrP^C^ signalosomes. Altered PTMs of PrP^C^ partners in prion-infected neurons affect the homeostatic activity of plasma membrane adhesion proteins, neuronal receptors, or ion channels, likely contributing to neuronal polarity and neurotransmission defects in prion diseases. Altered PTMs in PrP^C^ signalosomes impact downstream intracellular effectors such as Src kinases, ROCK, PDK1, PDK4, α-secretases, CREB transcription factor, and others. The imbalanced PTMs modify the biological activity or subcellular localization of these signaling effectors, thus hampering the signaling pathways they are involved in. It manifests by changes in redox equilibrium, metabolic reprogramming toward pro-oxidant fatty acids metabolism, high sensitivity to several stresses such as inflammation, and possibly autophagy derangement (López-Pérez et al., 2019), which compromise neuronal homeostasis and contribute to neurodegeneration in prion diseases. Most of the signaling effectors with disturbed PTMs listed in this review were identified with the help of prion-infected cell lines and primary neuronal cultures and confirmed *in vivo* in the brains of mouse models with prion-like diseases or even in the post-mortem brains of Creutzfeldt–Jakob disease patients. These signaling effectors currently represent attractive therapeutic targets to combat prion diseases and possibly other neurodegenerative diseases, such as Alzheimer’s and Parkinson’s diseases. Several studies have reported on the mechanistic convergence of these amyloid-based neurodegenerative diseases to PrP^C^. PrP^C^ displays the capacity to interact with several unrelated amyloid proteins, including PrP^Sc^, Alzheimer-linked Aβ oligomers, and Parkinson-linked pre-formed fibrils of pathological α-synuclein, and to relay their neurotoxicity (Laurén et al., 2009; Um et al., 2012; Aulić et al., 2017). Oligomers of Aβ or α-synuclein bind with a nanomolar affinity PrP^C^ at the same epitopes, including one epitope in the hinge region of PrP^C^ involved in PrP^C^ PTCC into PrP^Sc^ (Chen et al., 2010; Smith et al., 2019; Corbett et al., 2020; Scialò and Legname, 2020). It would be tempting to genetically modify those PrP^C^ epitopes with gene-editing technologies to generate PrP^C^ molecules that are unable to bind amyloids or to be converted into PrP^Sc^ with the perspective of limiting or canceling amyloid neurotoxicity. Because PrP^C^ is essential for the homeostasis of neurons and other cell types, such a genetic protective approach will be successful only if we are able to keep the PrP^C^ cell functions intact, including PrP^C^ signaling activity.

Of note, the binding to PrP^C^ of Aβ or α-synuclein does not promote the conversion of PrP^C^ into PrP^Sc^, as PrP^C^ PTCC into PrP^Sc^ is restricted to prion diseases. The binding to PrP^C^ of Aβ/α-synuclein oligomers would deregulate PrP^C^ signalosomes and downstream coupled signaling effectors on the removal of PrP^C^ from signalosomes (loss-of-PrP^C^ function). Alternatively, the binding to PrP^C^ of Aβ/α-synuclein oligomers would freeze PrP^C^ in signalosomes, maintaining PrP^C^ signalosomes in an active state (gain-of-PrP^C^ function). Whatever the scenario, the corruption of PrP^C^ signalosomes by PrP^Sc^, Aβ, or α-synuclein leads to post-translation modifications and deregulation of the same signaling effectors, such as ROCK, PDK1, and others (Pietri et al., 2013; Ferreira et al., 2017; Koch et al., 2018; Abd-Elrahman et al., 2020). In conclusion, common PTM patterns appear to contribute to neurodegeneration in prion diseases, Alzheimer’s diseases, and Parkinson’s diseases. However, the pending question remains as to which other specific PTM patterns can be specifically associated with each of these amyloid-based neurodegenerative diseases, as these diseases display different clinical manifestations.

## Author contributions

ChB: Writing – original draft, Writing – review & editing. ClB: Writing – original draft, Writing – review & editing. AB: Writing – original draft, Writing – review & editing. AA-B: Writing – original draft, Writing – review & editing. BS: Writing – original draft, Writing – review & editing. MP: Writing – original draft, Writing – review & editing.
